# Risk Factors and Incidence of Acute Ischemic Stroke: A Comparative Study Between Young Adults and Older Adults

**DOI:** 10.7759/cureus.14670

**Published:** 2021-04-24

**Authors:** Urvish K Patel, Mihir Dave, Anusha Lekshminarayanan, Preeti Malik, Matthew DeMasi, Sangeetha Chandramohan, Shreejith Pillai, Raghavendra Tirupathi, Shamik Shah, Vishal B Jani, Mandip S Dhamoon

**Affiliations:** 1 Public Health and Neurology, Icahn School of Medicine at Mount Sinai, New York, USA; 2 Internal Medicine, University of Nevada Reno, School of Medicine, Reno, USA; 3 Internal Medicine, Richmond University Medical Center, Staten Island, USA; 4 Rehabilitation Medicine, New York Medical College and Metropolitan Hospital Center, New York, USA; 5 Public Health, Icahn School of Medicine at Mount Sinai, New York, USA; 6 Neurology, Massachusetts General Hospital, Boston, USA; 7 Internal Medicine, Albert Einstein College of Medicine, Bronx, USA; 8 Internal Medicine, Henry Ford Health System, Detroit, USA; 9 Internal Medicine, Keystone Health, Chambersburg, USA; 10 Neurology, Stormont Vail Health, Topeka, USA; 11 Neurology, Creighton University School of Medicine, Omaha, USA; 12 Neurology, Icahn School of Medicine at Mount Sinai, New York, USA

**Keywords:** acute ischemic stroke, risk factors, young adults, nationwide inpatient sample (nis), ischemic cerebrovascular disease, hypercoagulable state, end stage renal disease, hiv, epilepsy, obesity

## Abstract

Introduction

Approximately 5-10% of strokes occur in adults of less than 45 years of age. The rising prevalence of stroke risk factors may increase stroke rates in young adults (YA). We aimed to compare risk factors and outcomes of acute ischemic stroke (AIS) among YA.

Methods

Adult hospitalizations for AIS and concurrent risk factors were found in the Nationwide Inpatient Sample database. Weighted analysis using chi-square and multivariable survey logistic regression was performed to evaluate AIS-related outcomes and risk factors among YA (18-45 years) and older patients.

Results

A total of 4,224,924 AIS hospitalizations were identified from 2003 to 2014, out of which 198,378 (4.7%) were YA. Prevalence trend of YA with AIS showed incremental pattern over time (2003: 4.36% to 2014: 4.7%; pTrend<0.0001). In regression analysis, the risk factors associated with AIS in YA were obesity (adjusted odds ratio {aOR}: 2.26; p<0.0001), drug abuse (aOR: 2.56; p<0.0001), history of smoking (aOR: 1.20; p<0.0001), infective endocarditis (aOR: 2.08; p<0.0001), cardiomyopathy (aOR: 2.11; p<0.0001), rheumatic fever (aOR: 4.27; p=0.0014), atrial septal disease (aOR: 2.46; p<0.0001), ventricular septal disease (aOR: 4.99; p<0.0001), HIV infection (aOR: 4.36; p<0.0001), brain tumors (aOR: 7.89; p<0.0001), epilepsy (aOR: 1.43; p<0.0001), end stage renal disease (aOR: 2.19; p<0.0001), systemic lupus erythematous (aOR: 3.76; p<0.0001), polymyositis (aOR: 2.72; p=0.0105), ankylosis spondylosis (aOR: 2.42; p=0.0082), hypercoagulable state (aOR: 4.03; p<0.0001), polyarteritis nodosa (aOR: 5.65; p=0.0004), and fibromuscular dysplasia (aOR: 2.83; p<0.0001).

Conclusion

There is an increasing trend in AIS prevalence over time among YA. Both traditional and non-traditional risk factors suggest that greater awareness is needed, with prevention strategies for AIS among young adults.

## Introduction

Stroke is the second leading cause of death globally, and although it is most common in the elderly, a significant number of young adults (YA) suffer from it every year [1,2]. The risk of stroke increases with age, but can occur at any age. In 2009, 34% of people hospitalized for stroke were less than 65 years old [3]. Approximately 5-10% of strokes occur in adults <45 years of age [4-8]. Despite considerable improvements in primary prevention, diagnostic workup, and treatment, stroke still remains a major cause of morbidity, serious physical and cognitive long-term disability, and loss in work-related productivity especially when it occurs in the younger population [9,10].

In a systematic review of stroke incidence in YA, the proportion of ischemic strokes ranged between 21.0% and 77.9% in patients under 45 years of age with first-ever stroke [11]. There were 59,077 deaths in YA in the United States from 1989 through 2009 due to stroke, contributing to 2868 deaths per year on average with an average annual mortality rate among YA being 0.93 per 100,000 persons for intracerebral hemorrhage (ICH), 1.1 per 100,000 persons for subarachnoid hemorrhage (SAH), and 0.70 per 100,000 persons for ischemic stroke [12]. In a single-center study comparing characteristics of stroke between younger and older patients, there were significant differences in risk factors, etiology, and distribution of sex between these groups [13]. Edwards et al. from the Canadian Institute for Health Information Discharge Abstract Database (n = 26,366) described a higher hazard for recurrent stroke at one year (hazard ratio {HR}: 6.8), at five years (HR: 5.1), stroke survivors had higher mortality and morbidity, and patients with TIA had a higher prevalence (31.5%; 1789/5677) of an adverse event within the first five years [14].

Our study aimed to provide estimates on the burden of stroke among YA in the United States. We performed a comprehensive assessment to compare traditional and non-traditional risk factors and ischemic stroke-related mortality, morbidity, discharge disposition, disability, and risk of death among young adults (YA: 18-45 years) vs. old adults (OA: >45 years) between 2003 and 2014.

## Materials and methods

Data were obtained from the Agency for Healthcare Research and Quality's Healthcare Cost and Utilization Project (HCUP) Nationwide Inpatient Sample (NIS) files from January 2003 to December 2014. The NIS is the largest publicly available all-payer inpatient care database in the United States and contains discharge-level data provided by states that participate in the HCUP (including a total of 46 states in 2011). This administrative dataset contains data on approximately eight million hospitalizations in 1000 hospitals that were chosen to approximate a 20% stratified sample of all US community hospitals, representing more than 95% of the national population. Discharge weights are provided for each patient discharge record, which helps to obtain national estimates. Each hospitalization is treated as an individual entry in the database and is coded with one principal diagnosis, up to 24 secondary diagnoses, and 15 procedural diagnoses associated with that stay (detailed information on NIS is available at http://www.hcup-us.ahrq.gov/db/nation/nis/nisdde.jsp). The NIS is a de-identified database, so informed consent or IRB approval was not needed for the study. The HCUP Data Use Agreement and training (HCUP-4Q28K90CU) for the data utilized in this study were obtained.

Study population

We used the ninth revision of the International Classification of Diseases, Ninth Revision, Clinical Modification (ICD-9-CM) codes to identify adult patients admitted with a primary diagnosis of AIS (ICD-9-CM codes 433.01, 433.11, 433.21, 433.31, 433.81, 433.91, 434.01, 434.11, 434.91). These codes have been previously validated and are 35% sensitive, 99% specific, with 96% positive predictive value (PPV), and 79% negative predictive value for the diagnosis of ischemic stroke [15]. We used ICD-9-CM codes to identify traditional and non-traditional risk factors. Table 1 lists all ICD-9-CM codes that were used for this study. Age <18 years and admissions with missing data for age, sex, and race were excluded.

**Table 1:** ICD-9-CM codes used in this analysis ICD9-CM: International Classification of Diseases, Ninth Revision, Clinical Modification; IHD: ischemic heart diseases; ASD: atrial septal disease; VSD: ventricular septal disease; PDA: patent ductus arteriosus; SLE: systemic lupus erythematosus; CNS: central nervous system; DM: diabetes mellitus; A-fib: atrial fibrillation; Hb SS: homozygous SCD patients; SC/HbC: sickle cell-hemoglobin C; SCD: sickle cell disease

Disease	ICD9-CM codes nationwide inpatient sample
Infective endocarditis	421.0
Cardiomyopathy	425.1 Primary CM, 425.20 Obscure cardiomyopathy of Africa, 425.30 Endocardial fibroelastosis, 425.40 Other primary cardiomyopathies, 425.50 Alcoholic cardiomyopathy, 425.70 Nutritional and metabolic cardiomyopathy, 425.80-429.83 Cardiomyopathy in other diseases classified Takotsubo, 425.90, 674.5, 414.8 Secondary cardiomyopathy, unspecified Peripartum cardiomyopathy Ischemic
Rheumatic fever	390, 391.9, 391.1, 391.8, 391.2, 391.0
IHD	410.00, 410.01, 410.02, 410.10, 410.11, 410.12, 410.20, 410.21, 410.22, 410.30, 410.31, 410.32, 410.40, 410.41, 410.42, 410.50, 410.51, 410.52, 410.60, 410.61, 410.62, 410.70, 410.71, 410.72, 410.80, 410.81, 410.82, 410.90, 410.91, 410.92, CAD: 414.00-414.07, old MI - 412
ASD	745.5, 745.61
VSD	745.4
PDA	747.0
Rheumatoid arthritis	714.0, 714.1, 714.2
Ankylosing spondylitis	720.0
Psoriatic arthritis	696.0
SLE	710.0
Scleroderma	701.0
Sjogren’s syndrome	710.2
Polymyositis	710.4
Dermatomyositis	710.3
Hypercoagulable disorders (factor V Leiden mutation, antiphospholipid antibodies, protein S deficiency, antithrombin III deficiency	286.53, 289.81, 795.79
Polycythemia rubra	238.4
Pneumonia	Viral - 480.0, 480.1, 480.2, 480.3, 480.8, 480.9, Pneumococcal- 481 Other - bacterial pneumonia- 482.0, 482.1, 482.2, 482.3, Strep - 482.30, 482.31, 482.32, 482.39, Staph - 482.40, 482.41, 482.42, 482.49, Other specified bacteria - 482.81, 482.82, 482.83, 482.84, 482.89, 482.9, 483.0, 483.1, 483.8 486
Urinary tract infection	599.0
TB meningitis	013.00
Tuberculoma	013.20
Neurosyphilis	094.0 Tabes dorsalis, 094.1 General paresis, 094.2 Syphilitic meningitis, 094.3 Asymptomatic neurosyphilis, 094.81 Syphilitic encephalitis, 094.82 Syphilitic parkinsonism, 094.83 Syphilitic disseminated, 094.84 Syphilitic optic atrophy, 094.85 Syphilitic retrobulbar neuritis, 094.86 Syphilitic acoustic neuritis, 094.87 Syphilitic ruptured cerebral aneurysm, 094.89 Other specified neurosyphilis, 094.9 Neurosyphilis, unspecified
Cryptococcal meningitis	321.0
Seizure	345.01 generalized nonconvulsive epilepsy 345.0 nonconvulsive/absence 345.1 Gen convulsion 345.2 Petit mal status 345.3 Grand mal status 345.4 Partial epi w impairment of consciousness 345.5 partial epi w/o impairment of cons 780.3 Convulsion excluding epileptic convulsion & of newborn 780.39 other convulsion 780.31 febrile convulsion 345.6 infantile spasms 345.81 Intractable epilepsy
CNS tumors	191.0, Cerebrum, except lobes and ventricles 191.1, Frontal lobe. 191.2, Temporal lobe 191.3, Parietal lobe. 191.4, Occipital lobe. 191.5, Ventricles, 191.6, Cerebellum, 191.7, Brain stem. 191.8, Other parts of brain, 191.9, Brain unspecified and cranial fossa unspecified.
AVM brain	747.81
Moyamoya	437.5
Giant cell arteritis	446.5
PAN	446.0
Takayasu's disease	446.7
Thromboangiitis obliterans	443.1
HIV	042, V08
Fabry's	272.7
Fibromuscular dysplasia	447.8 447.3
Sickle cell disease	282.60, 282.62 Hb SS with crisis 282.61 Hb SS without crisis 282.63 SC/HbC w/o crisis 282.64 SC/HbC w crisis 282.68 other SCD without crisis 282.69 other SCD with crisis 282.41 Sickle cell-thalassemia without crisis 282.42 Sickle cell-thalassemia with crisis
Pregnancy	V22.0, V22.1, V22.2, V23.9
Pregnancy-related conditions/complications	Hyperemesis 643.10, 643.11, 643.13 Preterm labor 644.00, 644.03, 644.10, 644.13, 644.20, 644.21 Antepartum hemorrhage, 641.10, 641.11, 641.13, 641.30, 641.31, 641.33, 641.80, 641.83, 641.90, 641.93 Preeclampsia and gestational hypertension 642.40, 642.41, 642.42, 642.43, 642.44, 642.50, 642.51, 642.52, 642.53, 642.54, 642.60, 642.61, 642.62, 642.63, 642.64, 642.70, 642.71, 642.72, 642.73, 642.74, 642.90, 642.91, 642.92, 642.93, 642.94, diabetes - 648.00, 648.01, 648.02, 648.03, 648.04, 648.80, 648.81, 648.82, 648.83, 648.84, Postpartum hemorrhage 666.00, 666.02, 666.04, 666.10, 666.12, 666.14, 666.20, 666.22, 666.24, 666.30, 666.32, 666.34, Puerperial septic thrombophlebitis: 670.30, 670.32, 670.34
Alcohol	303.00, 303.01, 303.02, 303.03 303.90, 303.91, 303.92, 303.93 305.0
substance abuse	305.90, 305.20, 305.21, 305.22, 305.23, 305.30, 305.31, 305.32, 305.33, 305.40, 305.41, 305.42, 305.43 305.50, 305.51, 305.52, 305.53 305.60, 305.61, 305.62, 305.63 305.70, 305.71, 305.72, 305.73 305.80, 305.81, 305.82, 305.83 305.90, 305.91, 305.392, 305.93
Smoking	305.1, V15.82
Hypertension	401.0, 401.9, Complications - 402.00, 402.10, 402.90, 403.00, 403.10, 403.90, 404.00, 404.10, 404.90, 404.01, 404.11, 404.91, 404.93, 404.13, 404.93
DM	250.00, 250.01, 250.02, 250.03, 250.10, 250.11, 250.12, 250.13, 250.20, 250.21, 250.22, 250.23, 250.30, 250.31, 250.32, 250.33, 250.40, 250.41, 250.42, 250.43, 250.50, 250.51, 250.52, 250.53, 250.60, 250.61, 250.62, 250.63, 250.70, 250.71, 250.72, 250.73,
A.fib	427.31
Hypercholesterolemia	272.0, 272.1, 272.2

Patient and hospital characteristics

Patient characteristics of interest were age, sex, race, insurance status, and concomitant diagnoses as defined above. The race was defined by white (referent), African American, Hispanic, Asian or Pacific Islander, and Native American. Insurance status was defined by Medicare (referent), Medicaid, Private Insurance, and Other/Self-pay/No charge. We defined the severity of co-morbid conditions using Deyo's modification of the Charlson Comorbidity Index (CCI) (Table 2).

**Table 2:** Deyo’s modification of CCI ICD9-CM: International Classification of Diseases, Ninth Revision, Clinical Modification; CCI: Charlson Comorbidity Index

Condition	ICD-9-CM codes	Charlson score
Myocardial infarction	410-410.9	1
Congestive heart failure	428-428.9	1
Peripheral vascular disease	433.9, 441-441.9, 785.4, V43.4	1
Cerebrovascular disease	430-438	1
Dementia	290-290.9	1
Chronic pulmonary disease	490-496, 500-505, 506.4	1
Rheumatologic disease	710.0, 710.1, 710.4, 714.0-714.2, 714.81, 725	1
Peptic ulcer disease	531-534.9	1
Mild liver disease	571.2, 571.5, 571.6, 571.4 –571.49	1
Diabetes	250-250.3, 250.7	1
Diabetes with chronic complications	250.4-250.6	2
Hemiplegia or paraplegia	344.1, 342-342.9	2
Renal disease	582-582.9, 583-583.7, 585, 586, 588-588.9	2
Any malignancy including leukemia and lymphoma	140-172.9, 174-195.8, 200-208.9	2
Moderate or severe liver disease	572.2-572.8	3
Metastatic solid tumor	196-199.1	6
AIDS	042-044.9	6

Outcomes

Our primary interest was to compare the prevalence of traditional and non-traditional risk factors of AIS among YA (18-45 years) and OA (>45 years). The secondary interest was to compare outcomes of AIS in YA and OA. The outcomes were all-cause mortality during hospitalization, morbidity (length of stay >10 days {>90th percentile of AIS hospitalization} and discharge to non-home {transfer to short-term hospital, skilled nursing facility, intermediate care facility, or home health care}), discharge disposition (discharge to home vs. non-home), All Patients Refined Diagnosis Related Groups (APR-DRG) risk of mortality, APR-DRG severity of illness (disability), length of stay (LoS), and cost of hospitalization [16]. APR-DRGs were assigned using software developed by 3M Health Information Systems, where score 1 indicates minor loss of function, 2-moderate, 3-major, 4-extreme loss of function or likelihood of death. APR-DRG coding system used in this study to assess the risk of mortality and severity of illness is externally validated. It is a reliable method with accurate and consistent results and is widely used by hospitals, consumers, payers, and regulators [17,18].

Statistical analysis

All statistical analyses were performed using the weighted survey methods in Statistical Analysis System (SAS) Version 9.4 (SAS Institute Inc., Cary, NC). Weighted values of patient-level observations were generated to produce a nationally representative estimate of the entire US population of hospitalized patients. Univariate analysis of differences between categorical variables (including demographics, comorbidities, risk factors, and concurrent conditions) and outcomes was tested using the chi-square test and analysis of differences between continuous variables (LoS and cost of hospitalization) was tested using unpaired student's t-test. Among AIS hospitalizations, the prevalence and mortality trends from 2003 to 2014 for YA and OA were tested and plotted using the Jonckheere trend test.

 In order to examine the relationship of age groups (YA vs. OA) with AIS-related risk factors and the relationship of age groups with AIS-related outcomes, we used mixed-effects multivariable survey logistic regression models. The models were weighted and adjusted for demographics (age, sex, race), patient-level hospitalization variables (admission day, primary payer, admission type, median household income category), hospital-level variables (hospital region, teaching versus nonteaching hospital, hospital bed size), comorbidities, traditional and non-traditional risk factors, and CCI in order to estimate the adjusted odds ratio (aOR) and 95% confidence interval (CI). Common conditions covered as risk factors and CCIs were adjusted only once in order to avoid over-adjustment. For each model, the c-index was calculated. All statistical tests used were two-sided, and p<0.05 was deemed statistically significant. No statistical power calculation was conducted prior to the study.

## Results

We have described prevalence trends and characteristics of AIS. We have also compared demographics, patient and hospital characteristics, comorbidities, and outcomes of AIS amongst YA and OA below.

Disease hospitalizations

**Figure 1:**
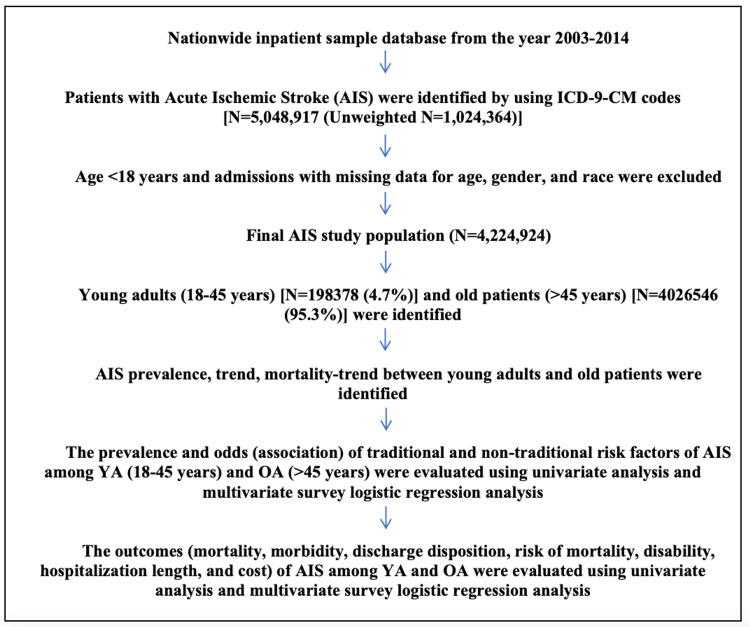
Flowchart detailing cohort selection and modeling analysis of outcomes ICD9-CM: International Classification of Diseases, Ninth Revision, Clinical Modification; AIS: acute ischemic stroke; YA: young adults; OA: old adults

**Figure 2:**
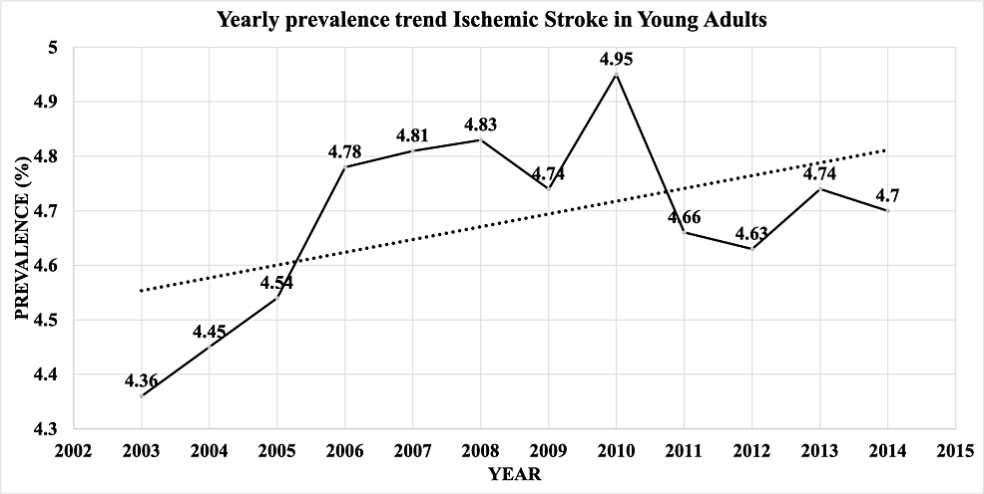
Yearly prevalence trend of AIS in Young adults AIS: acute ischemic stroke

There were 4,224,924 hospitalizations due to AIS from 2003 to 2014 after excluding patients with age <18 years and admissions with missing data for age, sex, and race (Figure 1). Out of 4,224,924 AIS hospitalizations, 198,378 (4.7%) were YA (≤45 years) and 4,026,546 (95.3%) were OA. As shown in Figure 2, the percentage of YA among AIS hospitalizations increased from 4.36% in 2003 to 4.7% in 2014. (pTrend<0.0001)

Demographics, patient and hospital characteristics, and comorbidities

There was a higher proportion of females among OA with AIS than YA (53.1% vs. 48.5%; p<0.0001) (Table 3). There was a higher proportion of African Americans (29.95% vs. 16.14%; p<0.0001) and Hispanics (12.17% vs. 7.35%; p<0.0001) among YA. Utilization of recombinant tissue plasminogen activator (6.32% vs. 4.34%; p<0.0001) and endovascular mechanical thrombectomy (1.22% vs. 0.58%; p<0.0001) were higher amongst YA with AIS compared to OA with AIS. OA with AIS had a higher prevalence of current long-term use of aspirin therapy compared to YA (8.79% vs. 5.12%; p<0.0001). Several co-morbidities like chronic blood loss anemia (0.7% vs. 0.45%; p<0.0001), liver disease (1.34% vs. 1.06%; p<0.0001), paralysis (5.48% vs. 3.7%; p<0.0001), psychosis (4.57% vs. 2.91%; p<0.0001) and chronic neurologic disorders (0.88% vs. 0.48%; p<0.0001) were higher among YA than OA. 

**Table 3:** Characteristics of AIS hospitalizations stratified by age group Percentage in brackets are column % indicates the direct comparison between young adults vs. older adults among patients with AIS. *This represents a quartile classification of the estimated median household income of residents in the patient's ZIP code. **Bedsize of hospital indicates the number of hospital beds which varies depends on hospital location (rural/urban), teaching status (teaching/non-teaching), and region (Northeast/Midwest/Southern/Western). CMV: cytomegalovirus; CNS: central nervous system; AIS: acute ischemic stroke

	Young adults (18-45 years)	Older adults (>45 years)	Total	p-Value
AIS hospitalizations (%)	198,378 (4.7)	4,026,546 (95.3)	4,224,924 (100)	
Demographics of patients				
Gender (%)				<0.0001
Female	96,233 (48.51)	2,138,020 (53.1)	2,234,253 (52.88)	
Male	102,145 (51.49)	1,888,457 (46.9)	1,990,602 (47.12)	
Race (%)				<0.0001
White	104,485 (54.54)	2,881,643 (73.4)	2,986,128 (72.53)	
African American	57,382 (29.95)	633,708 (16.14)	691,090 (16.78)	
Hispanic	23,310 (12.17)	288,462 (7.35)	311,772 (7.57)	
Asian or Pacific Islander	5158 (2.69)	103,386 (2.63)	108,544 (2.64)	
Native American	1233 (0.64)	18,600 (0.47)	19,833 (0.48)	
Characteristics of patients				
Median household income category for patient's ZIP code (%)*				<0.0001
0-25th percentile	66,609 (34.44)	1,175,425 (29.82)	1,242,034 (30.04)	
26-50th percentile	49,480 (25.58)	1,015,750 (25.77)	1,065,230 (25.76)	
51-75th percentile	42.785 (22.12)	920,665 (23.36)	963,450 (23.3)	
76-100th percentile	34,548 (17.86)	829,372 (21.04)	863,920 (20.89)	
Primary payer (%)				<0.0001
Medicare	18,906 (9.56)	2,804,887 (69.78)	2,823,793 (66.95)	
Medicaid	46,516 (23.52)	239,588 (5.96)	286,104 (6.78)	
Private insurance	87,892 (44.44)	711,337 (17.7)	799,229 (18.95)	
Other/self-pay/no charge	44,470 (22.48)	263,983 (6.57)	308,453 (7.31)	
Admission type (%)				<0.0001
Non- elective	190,181 (96.06)	3,833,377 (95.41)	4,023,557 (95.44)	
Elective	7806 (3.94)	184,578 (4.59)	192,384 (4.56)	
Admission day (%)				0.0018
Weekday	148,268 (74.74)	2,996,820 (74.43)	3,145,089 (74.44)	
Weekend	50,110 (25.26)	1,029,725 (25.57)	1,079,835 (25.56)	
Characteristics of hospitals				
Bedsize of hospital (%)**				<0.0001
Small	17,009 (8.63)	481,564 (12.01)	498,573 (11.85)	
Medium	47,805 (24.26)	1,030,840 (25.71)	1,078,644 (25.64)	
Large	132,226 (67.11)	2,497,587 (62.28)	2,629,813 (62.51)	
Hospital location & teaching status (%)				<0.0001
Rural	14,062 (7.14)	481,027 (12)	495,089 (11.77)	
Urban non-teaching	72,265 (36.68)	1,710,411 (42.65)	1,782,676 (42.37)	
Urban teaching	110,712 (56.19)	1,818,553 (45.35)	1,929,265 (45.86)	
Hospital region (%)				<0.0001
Northeast	37,829 (19.07)	858,527 (21.32)	896,356 (21.22)	
Midwest	32,990 (16.63)	697,196 (17.31)	730,186 (17.28)	
South	90,734 (45.74)	1,719,665 (42.71)	1,810,399 (42.85)	
West	36,826 (18.56)	751,158 (18.66)	787,983 (18.65)	
Stroke related medications (%)				
Current long-term use of Aspirin therapy	10,165 (5.12)	353,886 (8.79)	364,051 (8.62)	<0.0001
Use of recombinant tissue plasminogen activator (rtPA)	12,534 (6.32)	174,855 (4.34)	187,388 (4.44)	<0.0001
Use of endovascular mechanical thrombectomy	2413 (1.22)	23,411 (0.58)	25,824 (0.61)	<0.0001
Comorbidities of patients (%)				
Deficiency anemias	19,652 (9.94)	467,108 (11.65)	486,759 (11.57)	<0.0001
Rheumatoid arthritis/collagen vascular diseases	5321 (2.69)	94,806 (2.37)	100,127 (2.38)	<0.0001
Chronic blood loss anemia	1381 (0.7)	17,913 (0.45)	19294.6 (0.46)	<0.0001
Congestive heart failure	12,425 (6.28)	578,171 (14.43)	590,596 (14.04)	<0.0001
Chronic pulmonary disease	17,442 (8.82)	602,466 (15.03)	619,908 (14.74)	<0.0001
Hypothyroidism	8535 (4.32)	514,168 (12.83)	522,703 (12.43)	<0.0001
Liver disease	2654 (1.34)	42,508 (1.06)	45,162 (1.07)	<0.0001
Lymphoma	632 (0.32)	20,836 (0.52)	21,468 (0.51)	<0.0001
Fluid and electrolyte disorders	30,624 (15.49)	800,738 (19.98)	831,362 (19.77)	<0.0001
Metastatic cancer	1116 (0.56)	58,488 (1.46)	59,604 (1.42)	<0.0001
Paralysis	10,826 (5.48)	148,241 (3.7)	159,067 (3.78)	<0.0001
Psychoses	9036 (4.57)	116,737 (2.91)	125,774 (2.99)	<0.0001
Peptic ulcer disease excluding bleeding	25 (0.01)	1416 (0.04)	1441 (0.03)	<0.0001
Valvular disease	13,434 (6.8)	413,319 (10.31)	426,752 (10.15)	<0.0001
Weight loss	3289 (1.66)	125,944 (3.14)	129,233 (3.07)	<0.0001
Pulmonary circulation disorders	3317 (1.68)	115,370 (2.88)	118,687 (2.82)	<0.0001
Peripheral vascular disease	14,352 (7.26)	359,101 (8.96)	373,453 (8.88)	<0.0001
Coagulopathy	5614 (2.84)	110,457 (2.76)	116,071 (2.76)	0.0267
Solid tumor without metastasis	884 (0.45)	70,634 (1.76)	71,518 (1.7)	<0.0001
Depression	18,818 (9.52)	370,045 (9.23)	388,863 (9.25)	<0.0001
Other neurological disorders	1743 (0.88)	19,275 (0.48)	21,018 (0.5)	<0.0001
Concurrent conditions or risk factors (%)				
Diabetes	49,279 (24.84)	1,394,654 (34.64)	1,443,933 (34.18)	<0.0001
Hypertension (combined uncomplicated and complicated)	109,951 (55.42)	3,246,458 (80.63)	3,356,409 (79.44)	<0.0001
Obesity	31,797 (16.03)	306,293 (7.61)	338,091 (8)	<0.0001
Hypercholesterolemia	16,298 (8.22)	435,347 (10.81)	451,645 (10.69)	<0.0001
Drug abuse/dependence	21,714 (10.95)	67,645 (1.68)	89,359 (2.12)	<0.0001
Alcohol abuse/dependence	13,067 (6.59)	149,261 (3.71)	162,328 (3.84)	<0.0001
Past history of smoking	9262 (4.67)	379,316 (9.42)	388,579 (9.2)	<0.0001
Current tobacco dependence	61,720 (31.11)	574,046 (14.26)	635,766 (15.05)	<0.0001
Cardiac diseases	45,679 (23.03)	1,896,107 (47.09)	1,941,786 (45.96)	<0.0001
Ischemic heart disease	17,934 (9.04)	1,147,546 (28.5)	1,165,481 (27.59)	<0.0001
Infective endocarditis	965 (0.49)	6262 (0.16)	7227 (0.17)	<0.0001
Atrial Fibrillation	5926 (2.99)	947,502 (23.53)	953,428 (22.57)	<0.0001
Cardiomyopathy	10,203 (5.14)	144,888 (3.6)	155,091 (3.67)	<0.0001
Rheumatic fever	50 (0.03)	368 (0.01)	418 (0.01)	<0.0001
Rheumatoid heart disease	3859 (1.95)	133,365 (3.31)	137,224 (3.25)	<0.0001
Atrial septal disease	15,181 (7.65)	69,403 (1.72)	84,584 (2)	<0.0001
Ventricular septal disease	230 (0.12)	550 (0.01)	781 (0.02)	<0.0001
Patent ductus arteriosus	37 (0.02)	249 (0.01)	286 (0.01)	<0.0001
Infectious diseases	16,980 (8.56)	626,884 (15.57)	643,864 (15.24)	<0.0001
Urinary tract infection	11,209 (5.65)	509,319 (12.65)	520,528 (12.32)	<0.0001
HIV infection	2425 (1.22)	5679 (0.14)	8104 (0.19)	<0.0001
Pneumonia	4179 (2.11)	144,646 (3.59)	148,825 (3.52)	<0.0001
Neurosyphilis	133 (0.07)	1857 (0.05)	1989 (0.05)	<0.0001
CNS tuberculosis	16 (0.01)	25 (0)	41 (0)	<0.0001
Meningitis	75 (0.04)	276 (0.01)	352 (0.01)	<0.0001
CMV encephalitis	57 (0.03)	202 (0.01)	259 (0.01)	<0.0001
Toxoplasmosis	<10	0	<10	<0.0001
CNS lymphoma	<10	84	94	0.0142
Progressive multifocal encephalopathy	43 (0.02)	98	141	<0.0001
Non-infective CNS diseases	30,110 (15.18)	618,165 (15.35)	648,274 (15.34)	0.0355
Brain tumors	336 (0.17)	2442 (0.06)	2778 (0.07)	<0.0001
Epilepsy	16,641 (8.39)	235,995 (5.86)	252,636 (5.98)	<0.0001
Hemorrhagic stroke	3165 (1.6)	67,054 (1.67)	70,219 (1.66)	0.0172
Arterial-venous malformation	576 (0.29)	3919 (0.1)	4496 (0.11)	<0.0001
History of transient ischemic attack	11,304 (5.7)	338,425 (8.4)	349,728 (8.28)	<0.0001
Traumatic brain injury	217 (0.11)	7861 (0.2)	8078 (0.19)	<0.0001
Renal diseases	19,855 (10.01)	652,927 (16.22)	672,783 (15.92)	<0.0001
Chronic kidney diseases	7427 (3.74)	342,859 (8.51)	350,286 (8.29)	<0.0001
Acute renal failure	9328 (4.7)	279,229 (6.93)	288,557 (6.83)	<0.0001
End-stage renal disease	3787 (1.91)	61,935 (1.54)	65,722 (1.56)	<0.0001
Connective tissue diseases	5121 (2.58)	79,066 (1.96)	84,187 (1.99)	<0.0001
Systemic lupus erythematous	3959 (2)	14,170 (0.35)	18,129 (0.43)	<0.0001
Scleroderma	20 (0.01)	215 (0.01)	235 (0.01)	0.0051
Systemic sclerosis	230 (0.12)	3995 (0.1)	4226 (0.1)	0.0203
Rheumatoid arthritis	1022 (0.51)	59,416 (1.48)	60,438 (1.43)	<0.0001
Polymyositis	60 (0.03)	770 (0.02)	830 (0.02)	0.0007
Dermatomyositis	25 (0.01)	394 (0.01)	419 (0.01)	0.1855
Ankylosis spondylosis	91 (0.05)	971 (0.02)	1063 (0.03)	<0.0001
Psoriatic arthritis	124 (0.06)	2469 (0.06)	2593 (0.06)	0.8566
Coagulopathy	9792 (4.94)	34,353 (0.85)	44,145 (1.04)	<0.0001
Hypercoagulable state	9057 (4.57)	22,625 (0.56)	31,682 (0.75)	<0.0001
Polycythemia vera	784 (0.4)	11,921 (0.3)	12,705 (0.3)	<0.0001
Vasculitis	222 (0.11)	6278 (0.16)	6500 (0.15)	<0.0001
Giant cell arteritis	30 (0.01)	5576 (0.14)	5605 (0.13)	<0.0001
Polyarteritis nodosa	47 (0.02)	288 (0.01)	335 (0.01)	<0.0001
Takayasu disease	85 (0.04)	166 (0)	250 (0.01)	<0.0001
Thromboangiitis obliterans	66 (0.03)	248 (0.01)	314 (0.01)	<0.0001
Amyloidosis	19 (0.01)	3957 (0.1)	3976 (0.09)	<0.0001
Sickle cell disease	894 (0.45)	1772 (0.04)	2666 (0.06)	<0.0001
Moya-moya	1270 (0.64)	1020 (0.03)	2291 (0.05)	<0.0001
Fibromuscular dysplasia	560 (0.28)	2957 (0.07)	3517 (0.08)	<0.0001

Primary outcome

The prevalence of obesity (16.03% vs. 7.61%; p<0.0001), drug abuse (10.95% vs. 1.68%; p<0.0001), alcohol abuse (6.59% vs. 3.71%; p<0.0001), tobacco dependence (31.11% vs. 14.26%; p<0.0001), cardiomyopathy (5.14% vs. 3.6%; p<0.0001), atrial septal disease (7.56% vs. 1.72%; p<0.0001), epilepsy (8.39% vs. 5.36%; p<0.0001), and hypercoagulable state (4.57% vs. 0.56%; p<0.0001) were higher among YA in compare to OA.

The OA with AIS had higher prevalence of diabetes (34.64% vs. 24.84%; p<0.0001), hypertension (80.63% vs. 55.42%; p<0.0001), hypercholesterolemia/triglyceridemia (10.81% vs. 8.22%; p<0.0001) history of smoking (9.42% vs. 4.67%; p<0.0001), ischemic heart disease (28.5% vs. 9.04%; p<0.0001), atrial fibrillation (23.53% vs. 2.99%; p<0.0001), rheumatoid heart disease (3.31% vs. 1.95%; p<0.0001), urinary tract infection (12.65% vs. 5.65%; p<0.0001), pneumonia (3.59% vs. 2.11%; p<0.0001), history of transient ischemic attack (8.4% vs. 5.7%; p<0.0001), chronic kidney diseases (8.51% vs. 3.74%; p<0.0001), and acute renal failure (6.93% vs. 4.7%; p<0.0001).

Multivariable regression model derivation for the age-group specific risk factors

Table 4 shows multivariable models evaluating the odds of risk factors of AIS among YA and OA population. The obesity (aOR: 2.26; p<0.0001), drug abuse (aOR: 2.56; p<0.0001), past history of smoking (aOR: 1.20; p<0.0001), infective endocarditis (aOR: 2.08; p<0.0001), cardiomyopathy (aOR: 2.11; p<0.0001), rheumatic fever (aOR: 4.27; p=0.0014), atrial septal disease (aOR: 2.46; p<0.0001), ventricular septal disease (aOR: 4.99; p<0.0001), HIV infection (aOR: 4.36; p<0.0001), brain tumors (aOR: 7.89; p<0.0001), epilepsy (aOR: 1.43; p<0.0001), arterial-venous malformation (aOR: 1.81; p<0.0001), end-stage renal disease (aOR: 2.19; p<0.0001), systemic lupus erythematous (aOR: 3.76; p<0.0001), polymyositis (aOR: 2.72; p=0.0105), ankylosis spondylosis (aOR: 2.42; p=0.0082), hypercoagulable state (aOR: 4.03; p<0.0001), polyarteritis nodosa (aOR: 5.65; p=0.0004), and fibromuscular dysplasia (aOR: 2.83; p<0.0001) were significantly associated with YA population with AIS.

**Table 4:** Traditional and non-traditional risk factors of AIS among young adults in comparison to old adults aOR: adjusted odds ratio; CI: confidence interval; UL: upper limit, LL: lower limit; CNS: central nervous system; AIS: acute ischemic stroke

	Association of risk factors with young adults			Association of risk factors with old adults		
	aOR	95% CI (LL-UL)	p-Value	aOR	95% CI (LL-UL)	p-Value
Gender						
Female	Reference		<0.0001	Reference		<0.0001
Male	0.89	0.87-0.91		1.12	1.09-1.15	
Race						
White	Reference		<0.0001			<0.0001
African American	1.53	1.48-1.57		0.66	0.64-0.68	
Hispanic	1.47	1.42-1.53		0.68	0.65-0.71	
Asian or Pacific Islander	1.08	1.00-1.16		0.93	0.87-1.00	
Native American	1.27	1.08-1.48		0.79	0.67-0.93	
Median household income category for patient's ZIP code						
0-25th percentile	Reference		0.0002	Reference		0.0002
26-50th percentile	1.05	1.02-1.08		0.96	0.93-0.99	
51-75th percentile	1.01	0.98-1.04		0.99	0.96-1.03	
76-100th percentile	0.97	0.93-1.01		1.03	1.00-1.07	
Primary Payer						
Medicare	Reference		<0.0001	Reference		<0.0001
Medicaid	15.69	14.99-16.42		0.06	0.06-0.07	
Private insurance	11.10	10.69-11.53		0.06	0.09-0.09	
Other/self-pay/no charge	13.70	13.11-14.32		0.07	0.07-0.08	
Admission type						
Non-elective	Reference		0.0003	Reference		0.0003
Elective	0.90	0.84-0.95		1.12	1.05-1.19	
Admission day						
Weekday	Reference		0.1418	Reference		0.1418
Weekend	0.98	0.95-1.01		1.02	0.99-1.05	
Bedsize of hospital						
Small	Reference		<0.0001	Reference		<0.0001
Medium	1.16	1.11-1.21		0.86	0.82-0.90	
Large	1.25	1.20-1.30		0.80	0.77-0.78	
Hospital location & teaching status						
Rural	Reference		<0.0001	Reference		<0.0001
Urban non-teaching	1.16	1.11-1.20		0.86	0.82-0.90	
Urban teaching	1.34	1.28-1.41		0.74	0.71-0.78	
Hospital region						
Northeast	Reference		<0.0001	Reference		<0.0001
Midwest	1.16	1.11-1.20		0.87	0.83-0.90	
South	1.12	1.09-1.16		0.89	0.86-0.92	
West	1.02	0.98-1.06		0.98	0.94-1.02	
Concurrent conditions and risk factors						
Diabetes	0.71	0.69-0.74	<0.0001	1.40	1.36-1.45	<0.0001
Hypertension	0.32	0.32-0.33	<0.0001	3.09	3.01-3.17	<0.0001
Obesity	2.26	2.18-2.34	<0.0001	0.44	0.43-0.46	<0.0001
Hypercholesterolemia/triglyceridemia	0.80	0.77-0.84	<0.0001	1.24	1.19-1.30	<0.0001
Drug abuse	2.56	2.44-2.68	<0.0001	0.39	0.37-0.41	<0.0001
Alcohol abuse	0.75	0.71-0.78	<0.0001	1.34	1.28-1.41	<0.0001
Past history of smoking	1.20	1.17-1.24	<0.0001	0.83	0.81-0.86	<0.0001
Current tobacco dependence	0.64	0.61-0.67	<0.0001	1.56	1.48-1.65	<0.0001
Ischemic heart disease	0.46	0.44-0.47	<0.0001	2.20	2.11-2.28	<0.0001
Infective endocarditis	2.08	1.67-2.58	<0.0001	0.48	0.39-0.60	<0.0001
Atrial fibrillation	0.24	0.23-0.26	<0.0001	4.18	3.93-4.45	<0.0001
Cardiomyopathy	2.11	1.99-2.24	<0.0001	0.47	0.45-0.50	<0.0001
Rheumatic fever	4.27	1.76-10.36	0.0014	0.23	0.10-0.57	0.0014
Rheumatoid heart disease	0.86	0.78-0.95	0.0027	1.16	1.05-1.28	0.0027
Atrial septal disease	2.46	2.34-2.58	<0.0001	0.41	0.39-0.43	<0.0001
Ventricular septal disease	4.99	3.09-8.05	<0.0001	0.20	0.12-0.32	<0.0001
Patent ductus arteriosus	1.37	0.59-3.19	0.4681	0.73	0.31-1.70	0.4681
Urinary tract infection	0.64	0.61-0.67	<0.0001	1.56	1.49-1.64	<0.0001
HIV infection	4.36	3.62-5.26	<0.0001	0.23	0.19-0.28	<0.0001
Pneumonia	0.80	0.73-0.87	<0.0001	1.26	1.16-1.37	<0.0001
Neurosyphilis	0.62	0.38-1.00	0.0487	1.62	1.00-2.60	0.0487
Meningitis	1.93	0.84-4.45	0.1223	0.52	0.23-1.19	0.1223
CNS lymphoma	1.55	0.29-8.42	0.6096	0.64	0.12-3.49	0.6096
Brain tumors	7.89	5.48-11.36	<0.0001	0.13	0.09-0.18	<0.0001
Epilepsy	1.43	1.37-1.50	<0.0001	0.70	0.67-0.73	<0.0001
Hemorrhagic stroke	0.98	0.89-1.08	0.6444	1.02	0.93-1.13	0.6444
Arterial-venous malformation	1.81	1.43-2.28	<0.0001	0.55	0.44-0.70	<0.0001
History of transient ischemic attack	0.82	0.78-0.87	<0.0001	1.21	1.16-1.28	<0.0001
Traumatic brain injury	0.70	0.50-0.97	0.0328	1.43	1.03-2.00	0.0328
Chronic kidney diseases	0.63	0.57-0.71	<0.0001	1.58	1.42-1.75	<0.0001
Acute renal failure	0.84	0.79-0.89	<0.0001	1.19	1.12-1.26	<0.0001
End-stage renal disease	2.19	1.92-2.51	<0.0001	0.46	0.40-0.52	<0.0001
Systemic lupus erythematous	3.76	2.99-4.73	<0.0001	0.27	0.21-0.34	<0.0001
Scleroderma	2.25	0.50-10.18	0.2937	0.45	0.10-2.02	0.2937
Systemic sclerosis	1.18	0.79-1.77	0.4183	0.85	0.57-1.27	0.4183
Rheumatoid arthritis	0.44	0.34-0.56	<0.0001	2.29	1.79-2.93	<0.0001
Polymyositis	2.72	1.26-4.69	0.0105	0.37	0.17-0.79	0.0105
Dermatomyositis	1.06	0.39-2.91	0.9061	0.94	0.34-2.58	0.9061
Ankylosis spondylosis	2.42	1.26-4.69	0.0082	0.41	0.21-0.80	0.0082
Psoriatic arthritis	1.10	0.69-1.76	0.6998	0.91	0.57-1.46	0.6998
Hypercoagulable state	4.03	3.72-4.36	<0.0001	0.25	0.23-0.27	<0.0001
Polycythemia vera	1.10	0.91-1.33	0.3335	0.91	0.75-1.10	0.3335
Giant cell arteritis	0.24	0.11-0.54	0.0004	4.11	1.87-9.03	0.0004
Polyarteritis nodosa	5.65	2.16-14.81	0.0004	0.18	0.07-0.46	0.0004
Thromboangiitis obliterans	1.86	0.83-4.15	0.1304	0.54	0.24-1.20	0.1304
Amyloidosis	0.11	0.04-0.31	<0.0001	9.09	3.25-25.39	<0.0001
Fibromuscular dysplasia	2.83	2.20-3.65	<0.0001	0.35	0.27-0.45	<0.0001
Comorbidities of patients						
Deficiency anemias	1.15	1.11-1.20	<0.0001	0.87	0.83-0.90	<0.0001
Rheumatoid arthritis/collagen vascular diseases	0.96	0.77-1.19	0.6861	1.05	0.84-1.30	0.6861
Chronic blood loss anemia	1.82	1.56-2.13	<0.0001	0.55	0.47-0.64	<0.0001
Congestive heart failure	0.86	0.81-0.91	<0.0001	1.17	1.11-1.23	<0.0001
Chronic pulmonary disease	0.73	0.70-0.76	<0.0001	1.37	1.32-1.44	<0.0001
Hypothyroidism	0.55	0.52-0.58	<0.0001	1.83	1.73-1.93	<0.0001
Liver disease	0.76	0.68-0.85	<0.0001	1.32	1.18-1.47	<0.0001
Lymphoma	0.81	0.65-0.99	0.0410	1.24	1.01-1.53	0.0410
Fluid and electrolyte disorders	0.91	0.88-0.95	<0.0001	1.09	1.06-1.13	<0.0001
Metastatic cancer	0.38	0.32-0.45	<0.0001	2.63	2.23-3.12	<0.0001
Paralysis	1.45	1.36-1.53	<0.0001	0.69	0.65-0.73	<0.0001
Psychoses	1.48	1.39-1.57	<0.0001	0.68	0.64-0.72	<0.0001
Peptic ulcer disease excluding bleeding	0.43	0.16-1.17	0.0984	2.35	0.85-6.45	0.0984
Valvular disease	1.08	1.02-1.14	0.0090	0.93	0.88-0.98	0.0090
Weight loss	0.65	0.59-0.71	<0.0001	1.55	1.41-1.70	<0.0001
Pulmonary circulation disorders	0.90	0.82-0.99	0.0273	1.11	1.01-1.23	0.0273
Coagulopathy	1.05	0.97-1.13	0.2226	0.95	0.88-1.03	0.2226
Solid tumor without metastasis	0.22	0.18-0.26	<0.0001	4.65	3.81-5.68	<0.0001
Depression	1.14	1.09-1.19	<0.0001	0.88	0.84-0.92	<0.0001
Peripheral vascular disease	1.18	1.13-1.24	<0.0001	0.84	0.81-0.88	<0.0001
Other neurological disorders	1.25	1.08-1.44	0.0028	0.80	0.69-0.93	0.0028
Deyo-Charlson Comorbidity Index (CCI)	0.94	0.93-0.95	<0.0001	1.06	1.05-1.08	<0.0001
c-Index	0.898			0.898		

The odds of having diabetes (aOR: 1.40; p<0.0001), hypertension (aOR: 3.09; p<0.0001), hypercholesterolemia/triglyceridemia (aOR: 1.24; p<0.0001), alcohol abuse (aOR: 1.34; p<0.0001), current tobacco dependence (aOR: 1.56; p<0.0001), ischemic heart disease (aOR: 2.20; p<0.0001), atrial fibrillation (aOR: 4.18; p<0.0001), rheumatoid heart disease (aOR: 1.16; p=0.0027), urinary tract infection (aOR: 1.56; p<0.0001), pneumonia (aOR: 1.26; p<0.0001), history of transient ischemic attack (aOR: 1.21; p<0.0001), traumatic brain injury (aOR: 1.43; p=0.0328), chronic kidney diseases (aOR: 1.58; p<0.0001), acute renal failure (aOR: 1.19; p<0.0001), rheumatoid arthritis (aOR: 2.29; p<0.0001), giant cell arteritis (aOR: 4.11; p=0.0004), amyloidosis (aOR: 9.09; p<0.0001), solid tumor without metastasis (aOR: 4.65; p<0.0001) and with metastasis (aOR: 2.63; p<0.0001) were significantly higher among OA patients admitted with AIS. The c statistic was 0.898 (>0.5) which indicate good models.

Secondary outcomes

**Figure 3:**
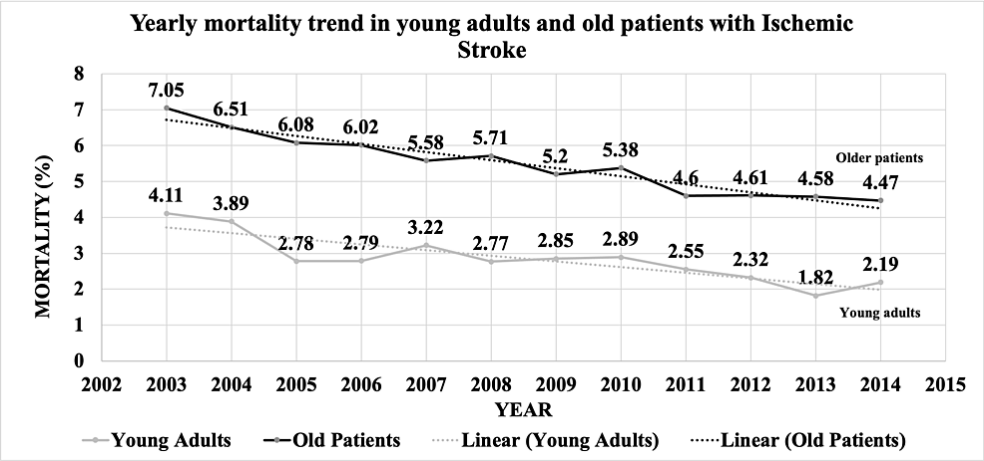
Yearly mortality trend in young adults and old adults with AIS AIS: acute ischemic stroke

Table 5 includes outcomes of AIS hospitalizations, comparing YA to OA. The all-cause in-hospital mortality (2.73% vs. 5.33; p<0.0001), morbidity (7.15% vs. 7.73; p<0.0001), major/extreme loss of function (30.7% vs. 37.21%; p<0.0001), and major/extreme likelihood of death (13.43% vs. 21.62%; p<0.0001) were lower among YA than OA AIS hospitalizations. YA AIS hospitalizations had a higher prevalence of discharge to home (64.59% vs. 36.15%; p<0.0001) than OA. The trend of all-cause in-hospital mortality in YA decreased from 4.11% in 2003 to 2.19% in 2014 (pTrend<0.0001) and decreased from 7.05% in 2003 to 4.47% in 2014 (P-Trend<0.0001) in OA AIS hospitalizations (Figure 3). Mean length of stay (5.6 days vs. 5.4 days; p<0.0001) and total cost of hospitalization were higher ($47,365 vs. $37,669; p<0.0001) in YA AIS hospitalizations than OA AIS hospitalizations (Table 5).

**Table 5:** Univariate analysis of outcomes among young vs. old adults with AIS hospitalizations Percentage in brackets are column % indicates the direct comparison between YA vs. old patients among patients with AIS. *Morbidity = length of stay >10 days (>90th percentile of AIS hospitalization) and discharge to non-home (transfer to short-term hospital, skilled nursing facility, intermediate care facility, or home health care). APR-DRG: All Patients Refined Diagnosis Related Groups; SNF: skilled nursing facility; ICF: intermediate care facility; SE: standard error; AIS: acute ischemic stroke

	Acute ischemic stroke hospitalizations			
	Young adults (18-45 years)	Older adults (>45-years)	Total	p-Value
All-cause in-hospital mortality (%)	5410 (2.73)	214,154 (5.33)	219,564 (5.21)	<0.0001
Morbidity* (%)	13,781 (7.15)	294,109 (7.33)	307,890 (7.70)	<0.0001
Discharge disposition (%)				<0.0001
Routine/home	121,623 (64.59)	1,364,958 (36.15)	1,486,581 (37.5)	
Transfer to short-term hospital	9593 (5.09)	117,357 (3.11)	126,950 (3.2)	
Transfer to SNF/ICF/another type of facility	42,160 (22.39)	1,772,822 (46.95)	1,814,981 (45.79)	
Home health care	14,913 (7.92)	520,542 (13.79)	535,455 (13.51)	
Total discharge other than home (%)	66,666 (35.41)	2,410,720 (63.85)	2,477,386 (62.5)	
APR-DRG severity/ loss of function (%)				<0.0001
Minor loss of function	34,616 (18.02)	425,141 (11.22)	459,756 (11.55)	
Moderate loss of function	98,510 (51.28)	1,953,248 (51.56)	2,051,758 (51.55)	
Major loss of function	47,274 (24.61)	1,173,725 (30.99)	1,220,999 (30.68)	
Extreme loss of function	11,687 (6.08)	235,835 (6.23)	247,522 (6.22)	
Total major/extreme loss of function (%)	58,961 (30.7)	1,409,560 (37.21)	1,468,522 (36.9)	<0.0001
APR-DRG risk mortality (%)				<0.0001
Minor likelihood of death	123,776 (64.44)	1,171,879 (30.94)	1,295,655 (32.55)	
Moderate likelihood of death	42,510 (22.13)	1,797,239 (47.45)	1,839,749 (46.22)	
Major likelihood of death	16,590 (8.64)	617,641 (16.31)	634,231 (15.94)	
Extreme likelihood of death	9210 (4.79)	201,191 (5.31)	210,401 (5.29)	
Total major/extreme likelihood of death (%)	25,800 (13.43)	818,832 (21.62)	844,632 (21.22)	<0.0001
Length of stay (mean) ± SE (days)	5.6 ± 0.04	5.4 ± 0.01		<0.0001
Cost of hospitalization (mean) ± SE ($)	47,365 ± 347	37,669 ± 58		<0.0001

Regression model derivation for the outcomes of YA

Table 6 includes multivariable regression analysis to predict outcomes of AIS among YA and OA population. All-cause in-hospital mortality (aOR: 0.56; 95%CI: 0.52-0.60), morbidity (aOR: 0.87; 95%CI: 0.83-0.91), discharge disposition to non-home (aOR: 0.60; 95%CI: 0.58-0.61), and major/extreme likelihood of death (aOR: 0.83; 95%CI: 0.81-0.86) were lower among YA than OA admitted with AIS with the c-statistic of 0.672, 0.690, 0.722, and 0.713, respectively (>0.5) which indicate good fit.

**Table 6:** Multivariable analysis of outcomes among young vs. old adults with AIS hospitalizations All models are adjusted for demographics (age, gender, race), patient-level hospitalization variables (admission day, primary payer, admission type, median household income category), hospital-level variables (hospital region, teaching versus nonteaching hospital, hospital bed size), comorbidities and risk factors like hypertension, diabetes mellitus, hypercholesterolemia, obesity, current smoking status, ex-smoker, drug abuse, alcohol abuse, and Charlson Comorbidity Index. *Morbidity = length of stay >10 days (>90th percentile of AIS hospitalization) and discharge to non-home (transfer to short term hospital, skilled nursing facility, intermediate care facility, or home health care) APR-DRG: All Patients Refined Diagnosis Related Groups; AIS: acute ischemic stroke

Odds ratio	95% confidence interval		p-Value	c-Index
	Lower limit	Upper limit		
All-cause in-hospital mortality in young adults (reference: older adults)				
0.56	0.52	0.60	<0.0001	0.672
Morbidity in young adults (reference: older adults)*				
0.87	0.83	0.91	<0.0001	0.690
Discharge disposition to non-home in young adults (reference: older adults)				
0.60	0.58	0.61	<0.0001	0.722
APR-DRG major/extreme loss of function in young adults (reference: older adults)				
1.02	0.998	1.05	0.0672	0.730
APR-DRG major/extreme risk of death in young adults (reference: older adults)				
0.83	0.81	0.86	<0.0001	0.713

## Discussion

We performed a population-based retrospective analysis of the nationally-representative NIS database to identify adult AIS hospitalizations and risk factors. Stroke in YA has been observed to be uncommon, and cerebrovascular disease reaches peak incidence later in life [19]. This observation has been confirmed in our study as we identified only 4.7% YA AIS hospitalizations, while 95.3% of hospitalizations were in patients who were 45 years or older. Thus, stroke is not a common health condition among YA. However, for those YA who do suffer a stroke, it is a considerable cause of morbidity and has an impact on the loss of work productivity in these patients [9]. Despite the small number of YA who suffer from stroke, we found an increasing prevalence among YA with AIS. From 2003 to 2014, hospitalizations for AIS in young adults increased from 4.36% to 4.70%. This stands in contrast to previous reports of stable rates of stroke incidence and decreasing rates of stroke hospitalization among adults [9]. A possible reason for this seemingly increasing incidence could be due to rising rates of stroke risk factors, including obesity, hypertension, diabetes, tobacco, and alcohol use [9].

Many risk factors among YA are traditional and modifiable, so screening and treatment are possible. These include obesity, drug abuse, history of smoking, infective endocarditis, cardiomyopathy, rheumatic fever, atrial septal disease, ventricular septal disease, HIV infection, and epilepsy. Some of the non-traditional risk factors like arterial-venous malformation, brain tumors, end-stage renal disease, systemic lupus erythematosus, polymyositis, ankylosis spondylosis, hypercoagulable state, polyarteritis nodosa, and fibromuscular dysplasia are significantly associated with YA with AIS.

Notably, in our study, all-cause in-hospital mortality was lower among YA. The prevalence rate of in-hospital mortality decreased from 2003 to 2014 (YA: 4.11% to 2.19% and OA: 7.05% to 4.47%), similar to Lee et al. (1998: 7.0% to 2007: 5.4%; p<0.0001) [20]. A possible explanation for this could be more effective treatment guidelines and strategies when presenting to the hospital. Young people may still be participating in high-risk factors that can lead to an increase in AIS hospitalizations, as shown in our study; however, treatment may have improved, with a concomitant decrease in mortality. Our study also indicated that YA with AIS hospitalizations had a lower chance of morbidity, discharge to short/long-term care, and the likelihood of death. YA and OA AIS hospitalizations had a similar mean LoS. However, the cost of hospitalizations was higher in YA ($47365 vs. $37669, p<0.0001). Stroke is thus an important cause of morbidity in young patients, and although having a small prevalence in the population, it affects hospitalization costs and dramatic impacts on quality of life in survivors. YA are also associated with higher long-term cumulative mortality due to stroke compared to the general population [21]. Stroke causes numerous physical and cognitive problems, long-term consequences, and work-related productivity losses especially in younger populations [21].

A major strength of this study was the findings that were nationally representative for the United States. However, there are limitations to this study. AIS was analyzed as a whole, rather than by identifying AIS patients according to subtype or by comparing other types of stroke. Perhaps, YA with AIS hospitalizations were due to a certain cause or presented as a subtype of AIS; however, this could not be elicited through this study. Additionally, being a retrospective study, we have associations between certain concurrent diagnoses and co-morbidities and AIS, yet we do not know if there is a temporal relationship between the two. We have evaluated in-hospital outcomes and do not have post-discharge records of these patients. Likewise, we are missing other details like stroke location, NIH Stroke Scale, concurrent medication use, the severity of risk factors, etc.

## Conclusions

AIS has been shown to be an uncommon problem in YA with better outcomes; however, with the rising prevalence trend of AIS over the past decade in young populations, prevention and treatment strategies need to be examined. Young adults have modifiable risk factors such as obesity, drug and smoking abuse, and heart conditions that can be screened. Besides that, non-traditional risk factors suggest that more awareness and prevention strategies can be targeted to the YA population. Further studies should be done to test whether modifying these factors lowers stroke risk in the young population or to determine if awareness campaigns differ based on the age of the patient targeted.
